# The combination of epigenetic drugs SAHA and HCI-2509 synergistically inhibits EWS-FLI1 and tumor growth in Ewing sarcoma

**DOI:** 10.18632/oncotarget.25829

**Published:** 2018-07-31

**Authors:** Daniel Jose García-Domínguez, Lourdes Hontecillas-Prieto, Pablo Rodríguez-Núñez, Guillem Pascual-Pasto, Monica Vila-Ubach, Rosa García-Mejías, María José Robles, Oscar M. Tirado, Jaume Mora, Angel M. Carcaboso, Enrique de Álava

**Affiliations:** ^1^ Institute of Biomedicine of Seville (IBiS), Hospital Universitario Virgen del Rocío/CSIC/Universidad de Sevilla/CIBERONC, Seville, Spain; ^2^ Developmental Tumour Biology Laboratory, Hospital Sant Joan de Déu, Barcelona, Spain; ^3^ Pathology Unit, Hospital Universitario Virgen del Rocío/CSIC/Universidad de Sevilla/CIBERONC, Seville, Spain; ^4^ Sarcoma Research Group, Laboratori d’Oncología Molecular, Institut d’Investigació Biomédica de Bellvitge (IDIBELL)/CIBERONC, L’Hospitalet de Llobregat, Barcelona, Spain

**Keywords:** Ewing sarcoma, EWSR1, epigenetic drug, synergism effect, PDX

## Abstract

**Purpose:**

Epigenetic regulation is crucial in mammalian development and maintenance of tissue-cell specific functions. Perturbation of epigenetic balance may lead to alterations in gene expression, resulting in cellular transformation and malignancy. Previous studies in Ewing sarcoma (ES) have shown that the Nucleosome Remodeling Deacetylase (NuRD) complex binds directly to EWS-FLI1 oncoprotein and modulates its transcriptional activity. The role of EWS-FLI1 as a driver of proliferation and transformation in ES is widely known, but the effect of epigenetic drugs on fusion activity remains poorly described. The present study evaluated the combination effects of the histone deacetylases inhibitor suberoylanilide hydroxamic acid (SAHA) and Lysine-specific demethylase1 inhibitor (HCI-2509) on different biological functions in ES and in comparison to monotherapy treatments.

**Results:**

The study of proliferation and cell viability showed a synergistic effect in most ES cell lines analyzed. An enhanced effect was also observed in the induction of apoptosis, together with accumulation of cells in G1 phase and a blockage of the migratory capacity of ES cell lines. Treatment, either in monotherapy or in combination, caused a significant decrease of *EWS-FLI1* mRNA and protein levels and this effect is mediated in part by fusion gene promoter regulation. The anti-tumor effect of this combination was confirmed in patient-derived xenograft mouse models, in which only the combination treatment led to a statistically significant decrease in tumor volume.

**Conclusions:**

The combination of SAHA and HCI-2509 is proposed as a novel treatment strategy for ES patients to inhibit the essential driver of this sarcoma and tumor growth.

## INTRODUCTION

Ewing sarcoma (ES) is the second most frequent sarcoma of bone and soft tissues in children and young adults. ES is characterized by translocations involving *EWSR1* and *ETS* transcription factors, with *EWS-FLI1* translocation being the most common and major driver for this disease [1, 2]. Despite significant therapeutic advances in multimodal therapy – including chemotherapy, surgery, and/or radiation – this aggressive tumor has poor survival rates. The disease-free survival (DFS) rate at 5 years remains below 70% in ES patients with localized disease, and ES patients with metastatic disease or relapse have an unfavorable prognosis with DFS rates below 30% [1]. Therefore, the identification of novel and more efficient therapeutic agents to reduce the toxicity and morbidity of treatment for curable tumors and improve survival in the metastatic/relapse settings is urgently required.

Cancer has traditionally been considered a multistep disease driven by the accumulation of mutations [3]. Nevertheless, massive sequencing analysis of ES tumors has revealed a remarkably stable genome at the time of diagnosis [4]. The most frequent gene mutations involve *STAG2* and *TP53,* and copy number alteration events such as gains of chromosome 1q, 2, 8 and 12, and losses of 9p (affecting *CDKN2A*) and 16q [4–7]. For this reason, researchers are seeking in the ES epigenome new alternatives to conventional strategies [1]. Recently, some publications have shed light on the role of the epigenome in oncogenesis and tumor progression in ES and have contributed to deeply understand how EWS-FLI1 participates in these processes [8–12]. Concerning the transcriptional function of EWS-FLI1, Sankar *et al.* described that the fusion protein can recruit transcriptional regulators like the Nucleosome Remodeling Deacetylase (NuRD) complex. This multiprotein complex is formed by different subunits including histone deacetylases (HDACs), lysine-specific demethylase 1 (LSD1) and others, which participate in the transcriptional regulation of EWS-FLI1 target genes [13].

HDACs and LSD1 are well-recognized players in the epigenetic regulation of cancer as well as emerging therapeutic targets [14]. On the one hand, HDACs are over expressed in different types of cancer [15–17] and thus have become a relevant target for epigenetic therapies. Specifically, the anticancer effect of the competitive pan-HDAC inhibitor suberoylanilide hydroxamic acid (SAHA) has been evaluated as a single agent *in vitro* in ES. These publications have shown that treatment with SAHA impairs ES cell growth and colony formation capacity; induces apoptosis, cell cycle alteration and DNA fragmentation; and increases the chemosensitivity of ES cells lines to standard treatment [18–20]. On the other hand, specific inhibitors of LSD1 have been proposed as a potential alternative due to the aberrant expression of LSD1 in several types of cancer such as breast [21, 22], colorectal [23], neuroblastoma [24], osteosarcoma, rhabdomyosarcoma, and synovial sarcoma [25, 26]. Specifically, HCI-2509, a reversible LSD1 inhibitor, disrupted the oncogenic activity of EWS-ETS fusions, impaired cell viability, and induced apoptosis as a single agent in ES cell lines [13, 25, 27, 28].

Since both LSD1 and HDACs have relevant roles in epigenetic regulation, the effectiveness of the combination of multiple HDAC and LSD1 inhibitors has been evaluated in several tumors. In glioblastoma, the combination *in vitro* showed an increment of apoptosis induction [29]. In primary acute myeloid leukemia, treatment increased apoptosis *in vitro* and improved the median survival of treated mice [30]. Finally, synergistic effects in apoptosis induction has been evaluated *in vitro* in rhabdomyosarcoma [31].

Since both LSD1 and HDACs are recruited by EWS-FLI1 to modulate its transcriptional activity, we considered that the inhibition of both NuRD subunits is a promising therapeutic preclinical study in ES. Indeed, we report a synergistic inhibition of the *EWS-FLI1* protein expression accompanied by synergistic effects on proliferation inhibition, strong migration impairment and apoptosis induction with the SAHA+HCI-2509 combination. Moreover, a reduction of tumor growth in ES patient-derived xenograft (ES-PDX) mouse models with this epigenetic drug combination was observed. We validated, for the first time, the potential efficacy of a combinatorial strategy using epigenetic drugs in ES.

## RESULTS

### SAHA and HCI-2509 combination synergistically inhibits proliferation in ES cell lines

To estimate SAHA and HCI-2509 ability to impair the growth *in vitro*, 12 ES cell lines were treated with either drug (Table 1). Median IC50 values at 72 h of treatment were obtained for SAHA and for HCI-2509. Proliferation assays showed that as single agents, SAHA and HCI-2509 inhibited the growth of ES cell lines. Specifically, the growth inhibition of HCI-2509 (IC50 = 0.267 µM) was more effective than that of SAHA (IC50 = 1.032 µM) as a single agent. Representative ES cells resistant and sensitive for both drugs were selected to study the effects of the SAHA+HCI-2509 combination *in vitro*. We calculated the IC50 SAHA/IC50 HCI-2509 ratio for each cell line (Table 2). We observed that the inhibition of cell growth was more effective for the combination of SAHA and HCI-2509 in six ES cell lines than that of single agents. We calculated the combination index (CI) and observed synergistic effects on proliferation inhibition in six out of the seven cell lines tested.

**Table 1:** HCI-2509 and SAHA inhibitory concentrations

			HCI-2509 (iLSD1)		SAHA	
Cell Line	*Fusion type*	*1q Copy number*	*IC50 (µM)*	*s.d.*	*IC50 (µM)*	*s.d.*
A4573	EWS-FLI1	Gain	0.247	0.025	0.673	0.086
A673	EWS-FLI1	Normal	0.097	0.053	1.676	0.347
CADO-ES	EWS-ERG	Gain	0.282	0.039	1.089	0.169
RDES	EWS-FLI1	Gain	0.485	0.095	0.867	0.145
RM82	EWS-ERG	Gain	0.534	0.079	1.261	0.135
SKNMC	EWS-FLI1	Normal	0.226	0.008	0.946	0.074
SK-ES-1	EWS-FLI1	Gain	0.351	0.010	1.262	0.140
STA-ET10	EWS-FEV	Gain	0.315	0.009	0.712	0.071
TC32	EWS-FLI1	Gain	0.187	0.019	1.553	0.161
TC71	EWS-FLI1	Gain	0.252	0.018	1.254	0.137
TTC466	EWS-ERG	Normal	0.251	0.013	0.765	0.148
WE68	EWS-FLI1	Gain	0.317	0.010	0.974	0.039

Proliferation IC50 of 12 ES cell lines assayed for HCI-2509 or SAHA sensitivity and measured after 72 h exposition to the drugs (*n* = 4).

**Table 2:** Combination assay: SAHA+HCI-2509

*Cell line*	*IC*_*50*_ *value ratio*	*Combination Index (CI)*	*Description*
**A4573**	2.7	**0.481** ± 0.13	Synergism
**A673**	17.3	**1.241** ± 0.10	Slight antagonism
**CADO-ES**	3.9	**0.775** ± 0.03	Moderate synergism
**SK-ES-1**	3.6	**0.514** ± 0.12	Synergism
**SK-N-MC**	4.2	**0.093** ± 0.02	Strong synergism
**TC32**	8.3	**0.393** ± 0.18	Synergism
**TTC466**	3.0	**0.592** ± 0.09	Synergism

CI values are mean ± s.d. (*n* = 3).

We further evaluated whether different ES gene fusion subtypes modulate sensitivity to these epigenetic drugs. There were no significant differences in IC50 values between cells bearing *EWS-FLI1* fusions with respect to those with other gene fusions (Supplementary Figure 1A and 1B). We also evaluated the impact of the gain of chromosome 1q on the response to both drugs since 1qG has been shown to have a strong negative impact on clinical outcome of ES patients [6]; no statistically significant differences were found (Supplementary Figure 1C and 1D). However, the 1qG cell lines showed a higher resistance to HCI-2509 treatment (Supplementary Figure 1C).

### SAHA, HCI-2509 and their combination promote cell cycle arrest and induce apoptosis in ES cell lines

**Figure 1:**
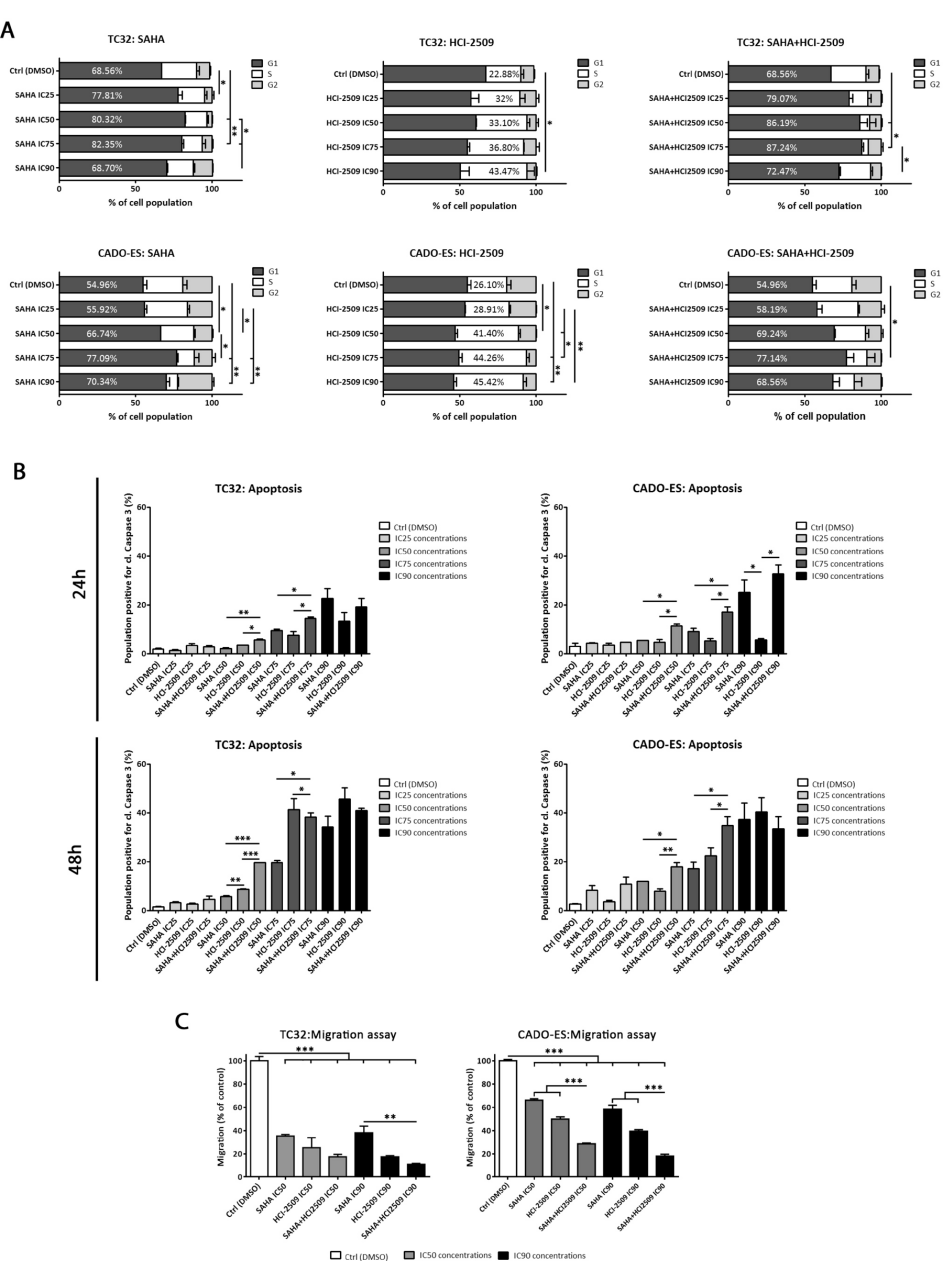
SAHA and HCI-2509 combination altered cell cycle progression, induced apoptosis and inhibited migration capacity *in vitro* (**A**) Distribution of cell cycle phases in TC32 and CADO-ES cell lines after 24 h of SAHA, HCI-2509 or combination treatment by flow cytometry. Percentages of the most affected phase are depicted in each condition. (**B**) Apoptosis induction analysis of population positive for cleaved Caspase 3 in TC32 and CADO-ES cell lines after 24h and 48h of SAHA, HCI-2509, alone or in combination, at different concentrations. (**C**) Migratory capacity analysis of transwell migration assay after 48 h of pre-treated TC32 and CADO-ES cell lines. Percentage of migratory cells is shown for each drug treatment respect to the control (DMSO) in both cell lines. All values show mean ± s.d. of three biological independent replicates. Statistical tests: significant analysis of variance, Tukey post-hoc test <0.001 (^***^), 0.01 (^**^) and 0.05 (^*^).

To understand the synergistic mechanisms involved in the inhibition of cell proliferation by the SAHA+HCl-2509 combination *in vitro*, we further investigated the effects of the combined treatment on cell cycle progression and apoptosis using two representative ES cell lines, TC32 and CADO-ES (CI values were near to the mean of ES cell lines analyzed). We evaluated the effects of SAHA, HCI-2509 or their combination at different concentrations on the cell cycle after 24 h of treatment (specific IC values for each cell line are shown in Supplementary Figure 1E). TC32 cells exposed to SAHA as a single agent were accumulated in the G1 phase compared to the control condition (Figure 1A). In contrast, TC32 cells exposed to HCI-2509 were arrested in S phase. Drug combination induced a strong delay in G1 (slightly higher proportion than SAHA monotherapy), with a concomitant decrease of cell populations in S phase. Similar effects were observed in the CADO-ES cell line (Figure 1A). To confirm that the G1 arrest of cells treated with the drug combination was mainly a consequence of SAHA, we compared the amount of cells in G1 in monotherapies and in combination. There was no difference in the G1 cell population in SAHA compared to the combination treatment in TC32 and CADO-ES cell lines (Supplementary Figure 2A).

To examine whether growth inhibition by SAHA+HCI-2509 combination was also attributable to programmed cell death, we studied apoptosis by cleaved Caspase 3. Both TC32 and CADO-ES cell lines were treated with single agents or in combination at low (IC25), medium (IC50, IC75) and high (IC90) concentrations at 24 h and 48 h. We demonstrated that SAHA and HCI-2509 (albeit to a lesser degree) as a single agents significantly induced apoptosis at high concentration after 24 h-48 h (Supplementary Figure 2B). Regarding combination treatment, we observed a significant difference in apoptotic induction with medium-high concentrations (IC75 and IC90) after 24 h of treatment and a significant difference in medium and high concentrations (IC50–IC90) after 48 h of treatment respect to the control and low concentrations (Supplementary Figure 2B). Finally, we compared the percentage of apoptosis induction between monotherapies and combination treatment (Figure 1B). We observed a significant higher apoptotic rate after combination therapy respect to any single agent in intermediate concentrations (IC50 and IC75). However, the combined IC90 concentration did not show such effect, probably because the maximum apoptotic effect had been reached for each drug (Figure 1B). These findings indicate that cell cycle arrest and apoptosis induction might be part of the mechanisms responsible for the observed cell proliferation inhibition by the SAHA+HCI-2509 combination treatment.

### Migratory capacity inhibition is enhanced by SAHA and HCI-2509 combination

The metastatic potential of tumors depends in part on the ability of tumor cells to migrate and invade distant sites. To investigate the effect of the epigenetic drugs on the mobility of ES cells *in vitro*, we used a well-established transwell migration assay.

Pre-treated TC32 and CADO-ES cell lines during 24 hours were evaluated for its migratory ability at 48 hours in drug-free conditions, to avoid by-stander effects, as apoptotic cells. A significant reduction in migratory capacity was found after SAHA or HCI-2509 treatment at IC50 and IC90 concentrations in both cell lines (Figure 1C). In addition, combination treatment statistically impaired migratory ability respect to the monotherapies in CADO-ES at IC50 and IC90. In TC32, a similar trend was observed, although only a statistically difference was found between combination treatment and SAHA at IC90.

### SAHA and HCI-2509 combination significantly inhibits EWS-FLI1 expression in ES cell lines

**Figure 2:**
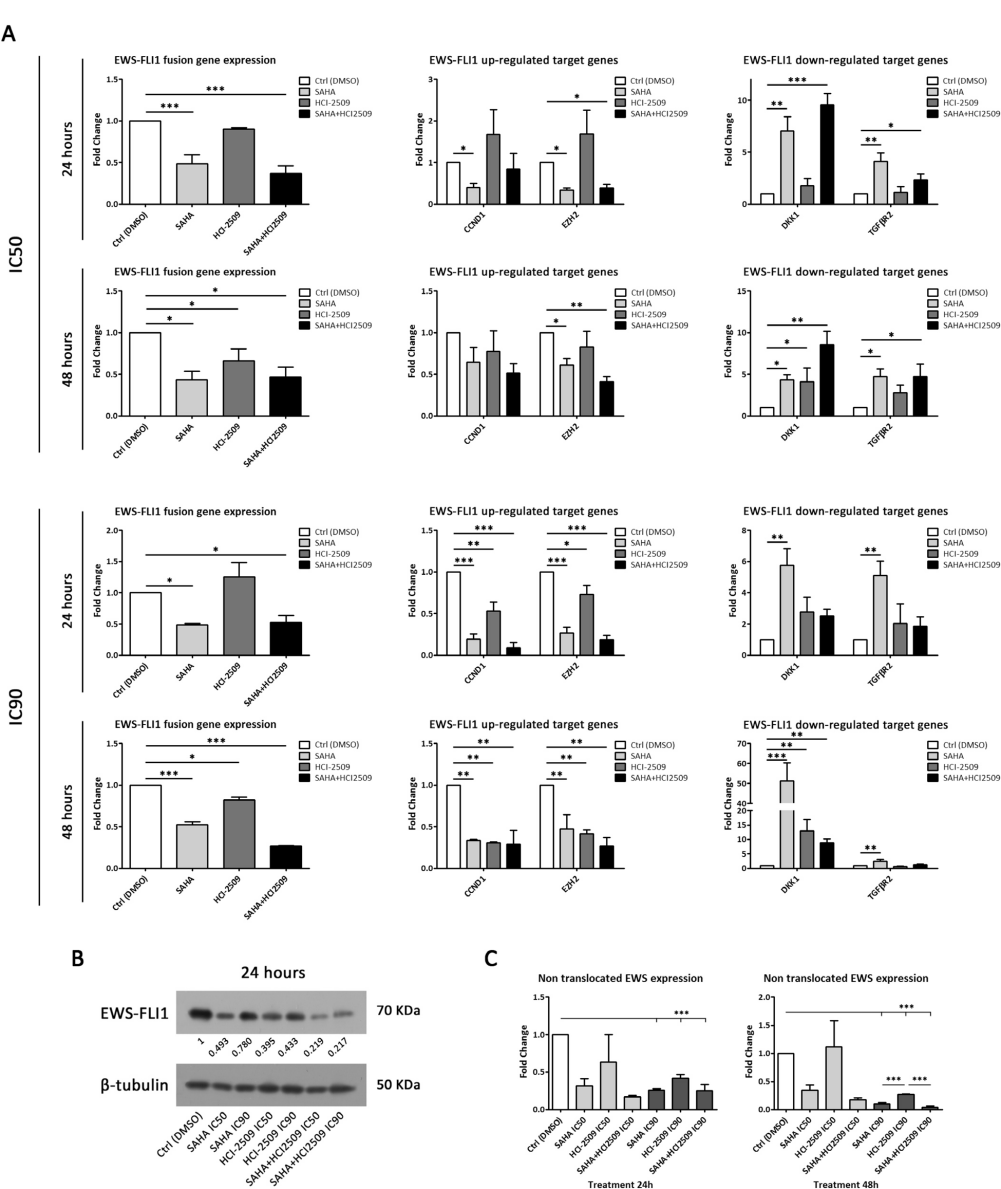
SAHA and HCI-2509 combination treatment inhibited EWS-FLI1 expression in TC32 cell line (**A**) mRNA expression analysis by RT-qPCR of EWS-FLI1 (left column), and EWS-FLI1-induced/repressed target genes (middle/right column) after 24 h and 48 h of SAHA, HCI-2509 and combination treatment at IC50 (upper panel) and IC90 concentrations (lower panel) in TC32 cell line. (**B**) Immunoblot of EWS-FLI1 protein expression after 24 h of SAHA, HCI-2509 and combination treatment at IC50 and IC90 concentrations in TC32 cell line. Relative quantification is shown respect to the control (DMSO). (**C**) mRNA expression analysis by RT-qPCR of non-translocated EWSR1 after 24 h (left panel) and 48 h (right panel) of SAHA, HCI-2509 and combination treatment at IC50 and IC90 concentrations in TC32 cell line. Mean ± s.d. of three biological independent replicates is shown. Statistical tests: significant analysis of variance, Tukey post-hoc test < 0.001 (^***^), 0.01 (^**^), and 0.05 (^*^).

We previously demonstrated that both monotherapies and combination treatments inhibited ES cell proliferation, but the mechanism by which SAHA and HCI-2509 can impair this process remains unknown. To this end, we analyzed the effect of these epigenetic drugs on EWS-FLI1 expression in EWS-FLI1-bearing TC32 and A673 cell lines. A 2-fold down-regulation of *EWS-FLI1* was observed under SAHA treatment compared with the control in all experimental conditions, while only a significant *EWS-FLI1* reduction after 48 h of HCI-2509 treatment was achieved at higher concentrations (IC90) in TC32 (Figure 2A). An enhanced effect in *EWS-FLI1* down-regulation was obtained after combination treatment in comparison with SAHA alone in both cell lines (Figure 2A and Supplementary Figure 3A).

To rule out that the *EWS-FLI1* down-regulation is due to a global transcriptional inhibition, we explored the expression of specific fusion target genes after drug treatment. EWS-FLI1 target genes expression was reverted after SAHA and SAHA+HCI-2509. We found a reduced expression of EWS-FLI1 up-regulated *CCND1* and *EZH2* target genes, while EWS-FLI1 down-regulated *DKK1* and *TGFBR2* target genes showed an increment of its expression (Figure 2A and Supplementary Figure 3A). Thus, a functional primary effect on *EWS-FLI1* expression is achieved by these epigenetic drugs. To explore whether a post-transcriptional mechanism is affecting fusion protein expression, we evaluated its levels after drug treatment at 24 h. As a common strategy in ES research, an anti-FLI1 antibody was used to specifically detect EWS-FLI1, since FLI1 is not expressed in ES cells [32]. Accordingly, to the down-regulation of the *EWS-FLI1* expression, a marked reduction in the fusion protein expression was observed after SAHA treatment. Surprisingly, a reduction of fusion protein expression was obtained with HCI-2509 as a single agent, although *EWS-FLI1* gene expression reduction was not observed with this drug. A significant effect on the EWS-FLI1 protein reduction was demonstrated after combination treatment in both ES cell lines (Figure 2B and Supplementary Figure 3B).

Thus, an enhanced effect on the reduction of EWS-FLI1, both at the mRNA and protein levels is demonstrated after SAHA+HCI-2509 treatment.

### *EWS-FLI1* promoter regulation contributes to SAHA+HCI2509-mediated EWS-FLI1 inhibition

Once we demonstrated *EWS-FLI1* transcriptional inhibition after drug treatment, we next searched for a possible molecular mechanism behind this effect. We evaluated if the *EWS-FLI1* promoter might be implicated in this regulation. Since the *EWS-FLI1* fusion retains the *EWSR1* promoter, *EWS-FLI1* promoter dependent-transcriptional regulation is analogous to that of endogenous *EWSR1* gene. For this reason, we evaluated *EWSR1* expression after drug treatments as an indirect approach. To assess this question, we used a probe that binds to exons 13–15 of *EWSR1* mRNA, which are transcribed only in the homologous non-translocated chromosome. Exons 13–15 of the *EWSR1* mRNA are under the regulation of FLI1 promoter in the translocated chromosome hence they are not expressed in ES cells [32].

A downregulation of *EWSR1* mRNA expression was observed after 24 h and 48 h drug treatments (IC50 and IC90) in TC32 cell lines with the exception of HCI-2509 monotherapy at IC50 concentration (Figure 2C). Moreover, a significant *EWSR1* down-regulation was observed with all drug treatments after 48h in the A673 cell line. Surprisingly, HCI-2509, alone or in combination, significantly induced an *EWSR1* mRNA increment at 24 h (Supplementary Figure 3C). This led us to consider that additional molecular mechanisms could be implicated in the maintenance of *EWSR1* mRNA induction after HCI-2509 treatment in A673 cell line.

Together, these results demonstrate a marked regulation of the *EWSR1* mRNA expression in the same way as *EWS-FLI1* mRNA regulation under these epigenetic treatments. Thus, we suggest that *EWS-FLI1* promoter regulation plays a role in epigenetic drug-mediated effect of fusion protein expression. This could explain why no differences of sensitivity to drug treatments used in this report, alone or in combination, because all ES cell lines analyzed share *EWSR1* as a partner in the different fusion types (Table 1 and Supplementary Figure 1A, 1B).

### Tumor growth is synergistically reduced by drug combination in ES PDX mouse models

PDX models have been found to be more predictive of patient responses to treatment than cell line derived-xenografts [33]. We performed survival studies in four ES PDX models: HSJD-ES-001, HSJD-ES-004, HSJD-ES-006 and HSJD-ES-011 after drug treatment. Safe doses were administered both for SAHA (100 mg/kg) and HCI-2509 (30 mg/kg) once daily (5 days on, 2 days off) intraperitoneal injection for three weeks. Weight loss was not observed in animals treated with SAHA and HCI-2509 as single agents or in combination. Histopathological evaluation did not reveal tissue damage in liver and lung (results not shown). However, localized intracellular vacuolization in renal tubular epithelial cells was observed after SAHA treatment, alone or in combination, after 21 days. This mild lesion was reverted after the end of drug treatment (Supplementary Figure 4A).

**Figure 3:**
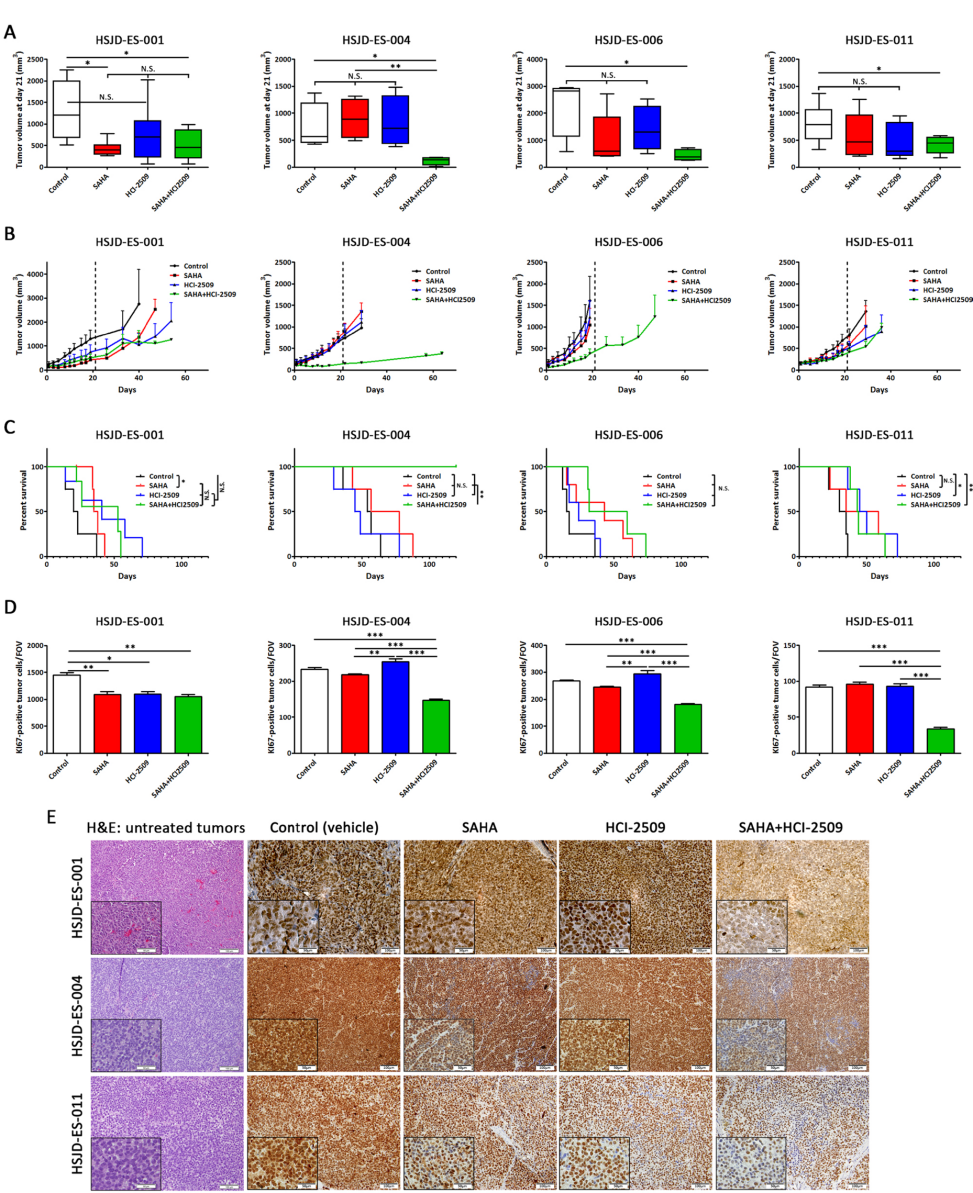
SAHA and HCI-2509 combination impaired tumor growth in ES PDX mice models (**A**) Tumor volumes (mm^3^) were measured after 21 days of SAHA, HCI-2509 and combination treatment in HSJD-ES-001, HSJD-ES-004, HSJD-ES-006, and HSJD-ES-011 PDX models. (**B**) Tumor growth was monitored in HSJD-ES-001, HSJD-ES-004, HSJD-ES-006, and HSJD-ES-011 PDX models upon single agent and combination therapies. Treatment was stopped after 21 days, and mice were followed until tumor volume reached 1,5 cm^3^. (**C**) Overall survival of HSJD-ES-001, HSJD-ES-004, HSJD-ES-006, and HSJD-ES-011 PDX mice treated with SAHA, HCI-2509, and its combination. Statistical tests: Log-rank (Mantel-Cox) test < 0.001 (^***^), 0.01 (^**^), and 0.05 (^*^). (**D**) Quantification of Ki67-positively labelled nuclei after 21 days of SAHA, HCI-2509, and combination treatment. Field of view (FOV). Statistical tests: significant analysis of variance, Tukey post-hoc test < 0.001 (^***^), 0.01 (^**^) and 0.05 (^*^). (**E**) Immunohistochemical staining of FLI1 in PDX tumor samples treated with SAHA and HCI-2509 alone or in combination after 21 days (20× and 40× magnifications). First column showed hematoxylin-eosin staining in untreated tumors.

Taking into account the tumor volume at the end of treatment (21 days), we observed that tumors in the combination groups were significantly smaller in the four models with respect to the control group (Figure 3A). The SAHA group was also significantly different from the combination group (in higher proportion that control-combination comparison) in the HSJD-ES-004 mice. Despite the differences in the tumor volume at day 21, tumors from HSJD-ES-001, HSJD-ES-006 and HSJD-ES-011 progressed after three cycles of treatment (Figure 3B). In HSJD-ES-004, combination therapy controlled tumor growth until the end of the study (120 days) and achieved a complete response in 25% of the animals (Figure 3B). Combination treatment dramatically improved overall survival of HSJD-ES-004 mice, the combination group extended survival up to a value greater than the evaluation period (120 days; *P* < 0.01). In the HSJD-ES-001 model, we observed an early significant difference between control and SAHA treatment. However, this subgroup had a dramatic decrease in survival thereafter. In the HSJD-ES-006 model, there was no statistically significant difference between control and treatment regimes. In the HSJD-ES-011 model, HCI-2509 and combination treatments led to a statistically significant increase in survival (Figure 3C). Drug treatments modulated Ki67 nuclear expression (Supplementary Figure 4B). In all models, in comparison with control condition, there was a significant decrease of Ki67 positive cells only after the combination treatment (Figure 3D). Besides, in the HSJD-ES-001 model, we observed a significant decrease after monotherapy treatments.

To confirm the drug-mediated effect on the main driver of ES, EWS-FLI1 protein expression was evaluated in PDX tumor samples. A subgroup of mice from each experimental condition was sacrificed and tissue samples were analyzed by immunohistochemistry after 21 days of treatment (Figure 3E). Histopathological evaluation revealed conventional appearance of ES as a neoplasm made up of small round cells (hematoxylin-eosin staining) and that only ES tumor cells were positive for FLI1 staining. A slight decrease of EWS-FLI1 expression was observed after monotherapy treatments compared to the control in HSJD-ES-004 and HSJD-ES-011 models. Moreover, a synergistic reduction of EWS-FLI1 was observed in cases treated with the drug combination compared to any other experimental condition. In addition, we found wide areas with almost complete loss of EWS-FLI1 expression after combination treatment. We suggest that these areas are more accessible to drug exposition or correspond to a more sensitive subpopulation. According to EWS-FLI1 down-regulation, fusion-induced targets (CCND1 and EZH2) and fusion-repressed target (TGFβR2) were down- and up-regulated, respectively, as expected (Supplementary Figure 4C).

Overall, the ES PDX *in vivo* assays demonstrated that the combination of SAHA with HCI-2509 was more effective than monotherapy treatment for tumor growth inhibition in ES.

## DISCUSSION

Conventional multimodal treatment regimens in ES have achieved remarkable improvements in survival over the last 30 years [1]. Despite therapeutic advances, for nearly all ES patients who relapse or develop metastasis, the therapeutic benefit of chemotherapy has reached a plateau [34]. One therapeutic strategy to move this field forward might involve epigenetic approaches that constitute a promising research area in ES [14, 35].

The first generation of epigenetic drugs, such as SAHA, has shown modest anti-tumor efficacy in Phase I and II clinical trials in patients with solid tumors in monotherapy [35], as well as in acute myeloid leukemia [36]. Thus, new strategies are based on the use of second generation of epigenetic drugs (greater selectivity for their molecular targets, i.e. HCI-2509), the combination of both or their combination with conventional drugs (ClinicalTrials.gov NCT00106626 or NCT00691210). Regardless, the epigenetic therapy field remains poorly explored in ES.

Our results showed that ES cell lines were sensitive to proliferation inhibition with first-generation SAHA (median IC50 = 1.032 µM) and, more efficiently, with second-generation HCI-2509 in monotherapy (median IC50 = 0.267 µM). We demonstrated a synergistic effect in the inhibition of proliferation in combination, with CI values between moderate and strong synergism in the majority of ES cells analyzed. These results confirm the superiority of SAHA+HCl-2509 combination in comparison to the monotherapy treatments in ES, as in other kind of tumors [29–31].

It has been described that the balance of cell proliferation and cell death must be regulated to maintain the control of tumor growth and many studies suggest that this regulation may be achieved, in part, regulating cell cycle progression [37]. For that reason, we explored the *in vitro* effects of the combined treatment on cell cycle progression and apoptosis. We observed that SAHA, alone or in combination, induced G1 phase delay, while S phase delay was observed with HCI-2509 treatment. Predominant effect of SAHA in the combination could be explained by the fact that SAHA-affected phase is previous to HCI-2509-affected phase. In addition, apoptosis induction was significantly higher in combination compared to monotherapies. This effect has been reported in many preclinical studies, including sarcomas, with alternative combinations of HDACs and LDS1 inhibitors [29–31]. Concerning migration, the higher motility impairment in monotherapy was found after HCI-2509 treatment. Nevertheless, a deeper migration inhibition was observed after combination treatment respect to monotherapies, probably because SAHA enhances the effect of LSD1 inhibitor. It is known that angiogenesis-related genes and cell invasion and migration are enhanced by HDACs [38]. Consistent with our results, a SAHA-induced cell migration inhibition effect was demonstrated in ovarian [39] and pancreatic cancer [40].

An essential epigenetic regulation has been described for EWS-FLI1, the main driver of the disease. We next investigated the effect of epigenetic drugs on EWS-FLI1 expression and its target genes. Intriguingly, we found that EWS-FLI1 expression was inhibited, both at mRNA and protein levels, after monotherapy treatments. Inhibition of *EWS-FLI1* transcript after combination treatment was similar or slightly higher than SAHA as a single agent, suggesting that SAHA plays a predominant role within this combination. Recently, Souza *et al.* have demonstrated that sodium butyrate (a potent class I and IIa HDAC inhibitor) induced suppression of cell proliferation accompanied by reduced transcriptional expression of the EWS-FLI1 [41]. A stronger effect on the inhibition of EWS-FLI1 protein was found with combination treatment in comparison to monotherapies. The specific inhibition of EWS-FLI1 protein was confirmed by the restoration of expression of EWS-FLI1 up- and down-regulated target genes after monotherapy and combination treatments. Theoretically, if both up and down-regulated genes recovery were only an effect of drug-mediated EWS-FLI1 inhibition, similar levels would then be found. However, recovery of down-regulated genes was found to be higher than up-regulated genes. As LSD1 and HDACs, molecular targets of our epigenetic approach, are effectors subunits of the NuRD complex, this differential effect could be explained by the intrinsic transcriptional repressor nature of the NuRD complex [13].

With the attempt to elucidate the mechanism of EWS-FLI1 expression after epigenetic drug treatment, we analyzed the expression of the non-translocated *EWSR1* gene since the promoter region is shared. We observed an analogous reduction of *EWSR1* and *EWS-FLI1* gene expression after treatments suggesting that this regulation in fact involves a promoter based mechanism. Nevertheless, after a 24 h-treatment with HCI-2509, alone and in combination, an increment of *EWSR1* expression together with an inhibition of *EWS-FLI1* was observed in the A673 cell line. Therefore, we reasoned that the aforementioned inhibition of the fusion protein could be mediated both by promoter regulation and by other unknown molecular mechanisms.

ES-PDX mouse models were used to evaluate our results *in vivo*. Reduction of tumor growth was not observed in monotherapy treated mice (except at early time of treatment with SAHA alone in HSJD-ES-001 model). However, inhibition of tumor growth was observed by the SAHA+HCl-2509 combination in four different ES-PDX models. A previous preclinical report in ES showed growth tumor reduction after SAHA [42] or HCI-2509 [27] as single agents. These conflicting results could be explained because ES cell line-derived xenograft models were used. PDXs are considered clinically more relevant as preclinical models [43, 44] because tumors retain some of the original characteristics, such as heterogeneity, and thus, response of PDX to drug treatments is closer to that in ES patients. We confirmed an *in vivo* down-regulation of EWS-FLI1 protein expression after combination treatment. Franzetti *et al.* recently proposed a model in which EWS-FLI1 low levels drive ES cells dissemination [45]. SAHA+HCI-2509-mediated tumor fusion inhibition did not result in an increment of the metastasis incidence. Histopathological evaluation discarded macroscopic lung metastatic lesions after drug treatment (21 days) and increased dissemination was not observed in long-term alive HSJD-ES-004 mice (120 days).

Recent clinical studies have demonstrated that combination of HDACs inhibitors with standard drug regimens represents a potentially effective therapeutic strategy [46, 47]. Along the same lines, we propose SAHA+HCI-2509 as a complement to the current multimodal treatments in ES since the preclinical work here disclosed shows promising anti-ES activity with tolerable toxicity profile.

## MATERIALS AND METHODS

### Cultured cell lines and pharmaceutical compounds

A4573, A673, CADO-ES, RDES, RM82, SK-ES-1, SK-N-MC, STAET10, TC32, TC71, TTC466, and WE68 were obtained from ATCC and the EuroBoNet cell lines panel, which is maintained and regularly checked and characterized by Ottaviano *et al.* [48] in Heinrich-Heine-University, Düsseldorf, Germany. Cells were grown on 0.1% gelatin-coated plates in RPMI 10% FBS except for A673 (DMEM 10%), SK-ES-1 (McCoy’s 15%), SK-N-MC (EMEM 10%) and RDES (RPMI 15%). Cells were maintained in 37° C incubators, in an atmosphere of 5% CO_2_. All cells were free of mycoplasma, as screened with the MycoAlert^®^ Mycoplasma Detection Kit (Lonza).

SAHA and HCI-2509 were purchased from Tocris Bioscience (UK) and Xcess Biosciences Inc. (US) respectively. Stock solutions of both compounds were prepared in dimethyl sulfoxide (DMSO) and diluted to final concentration in the culture medium 1:1000 (v/v).

### Cell viability assays

SAHA and HCI-2509 were added to complete growth medium at concentrations ranging from 0.01 to 100 µM to calculate the IC50 values in monotherapy. SAHA+HCI-2509 combination was added to complete growth medium at concentrations ranging from 0.0003 to 10µM (maintaining the IC50 values ratio between the two drugs). After 72 h, cells were subjected to ATP-lite assay (PerkinElmer, Waltham, MA, USA) and inhibitory concentrations were calculated using CalcuSyn software Version 2.0 (Biosoft). Combination index (CI) values were based on the mean growth inhibitions of SAHA and HCI-2509 in monotherapy and in combination. CI was calculated according to the Chou-Talalay method [49]. Synergy levels can be broadly divided into : <0.1, very strong synergism; 0.1–0.9, synergism (ranging from strong synergism to slight synergism); and 0.9–1.1, nearly additive to additive.

### Flow cytometry analyses

Cell flow cytometry analyses were conducted to evaluate cell cycle and apoptosis. TC32 and CADO-ES cell lines were exposed to 24 h and 48 h drug treatments. Non-confluent cultures of exponentially growing cells were trypsinized and ethanol fixed. To measure apoptosis, anti-Cleaved Caspase 3 (Asp175) (D3E9) (Cell Signaling #9603) was added in 0.5% BSA, 0.5% Triton X-100 PBS at 1:200. Alexa Fluor 488-labeled secondary antibody (1:10000; Invitrogen #A11008) was applied after washes. The antibody was incubated for 1 h at room temperature; controls lacking primary antibodies were done in parallel. Next, cells lines were incubated in PBS containing propidium iodide and RNAse A for 2 h. Flow cytometry data was processed and analyzed with FlowJo software (Tree Star). The sub-G1 population was gated out to improve the calculation of cell percentages at every cell cycle stage by built-in software algorithms.

### Transwell migration assay

A migratory assay was carried out to determine the migration ability of TC32 and CADO-ES cell lines. Transwell migration assays were carried out using inserts of polycarbonate membrane with 8μm pore size (Thermo Scientific, #140629). Cells were previously treated 24 h with SAHA, HCI-2509 or the combination at IC50 and IC90 concentrations. After drug removal, cells were harvested (3 × 10^5^ cells/well) in serum-free medium to triplicate wells of boyden chambers. 10% FBS-containing medium was added to the lower chamber as a chemo-attractant. After 48 h, upper inserts were washed three times, dried at room temperature, fixed with cold methanol for 10 min, and stained with DAPI (Sigma D-9542) for 10 min. Migratory cells was photographed under the inverted Leica microscope (Leica Microsystems). Seven random fields from each of the triplicate migration assays were counted.

### mRNA expression analysis

The expression of selected genes was analyzed by qRT-PCR. RNA was isolated from TC32 and A673 ES cell lines using miRVana miRNA Isolation Kit (Ambion; Life Technologies, USA). The quantity and quality of the total RNA was determined with Nanodrop ND-2000 Spectrophotometer (Thermo Scientific). Prior reverse transcription was performed using TaqMan Reverse Transcription Kit (Applied Biosystems; Life Technologies) in GeneAmp PCR 9700 thermocycler and qRT-PCR amplification with TaqMan Universal PCR Master Mix (Applied Biosystems). All qRT-PCR measurements were obtained in a 7900HT Fast Real Time PCR System with ExpressionSuite Software v1.0 (Applied Biosystems). Taqman probes utilized in this study are listed in Supplementary Table 1.

### Protein extraction and Western blot

Proteins were extracted from TC32 and A673 ES cell lines in RIPA buffer (150 mM NaCl, 1% (v/v) NP40, 50 mM Tris-HCl pH 8.0, 0.1% (v/v) SDS, 1mM EDTA, and 0.5% (w/v) deoxycholate) supplemented with protease inhibitor, 10 mM NaF and 2 mM NaOv. Immunoblotting was performed using the following antibodies: EWS-FLI1 expression was determined using the anti-FLI1 antibody (C-19) (Santa Cruz, #sc-356) overnight at 1:1000 dilution, followed by anti-rabbit IgG, HRP (Cell Signaling, #7074) for 1h at 1:10000; and calnexin (E-10) (Santa Cruz, #sc-46669) overnight at 1:1000 dilution, followed by anti-mouse IgG-HRP (Cell Signaling, #7076) for 1h at 1:10000. Protein bands were visualized using the Clarity Western ECL Substrate chemiluminescence detection kit (Bio-Rad, #170-5060). ImageJ software was applied for densitometric quantifications.

### *In vivo* preclinical testing in ES PDX models

Four ES PDX models established from patient biopsies at Sant Joan de Déu Hospital (HSJD, Barcelona, Spain) were used for the *in vivo* experiments. Two of these models (HSJD-ES-004 and HSJD-ES-006) have already been detailed in previous studies [50]. The clinical characteristics of the ES patients are included in Supplementary Table 2.

Athymic nude mice bearing 100–500 mm^3^ tumors in both flanks were randomized in 4 groups so that there were six tumors included in each group. One group received an intraperitoneal injection (IP) of 100 mg/kg SAHA once daily (5 days on, 2 days off) for three weeks; a second group was treated with an IP of 30 mg/kg HCI-2509 once daily (5 days on, 2 days off) for three weeks; a third group was treated with the combination of SAHA and HCI-2509 under the same regimens; and a fourth group was not treated (control). SAHA was first diluted with DMSO and then diluted 1:5 with 20% HPBCD in PBS. HCI-2509 was diluted to the appropriate concentration with DMSO. To study the activity of the different regimens, we evaluated tumor response at the end of treatment (day 21) and animal survival until the end of the study (day 120). One animal from each group was sacrificed at day 21 to collect tumor samples after treatment. Animal survival was defined as the time interval between the initial date of treatment and the date on which the threshold 1.5 cm^3^ tumor volume was reached. These experiments were carried out with the approval by the local animal care and use committee animal protocol number HSJD 135/11.

### Immunohistochemistry

Tumors and whole organs (kidney, liver, and lung) excised from the sacrificed mice were immediately formalin-fixed and paraffin-embedded. Representative sections were incubated with primary antibodies overnight at 4° C (1:100): anti-Ki-67 rabbit monoclonal antibody (clone 30-9, Roche) and anti-Fli-1 (MyBiosource, #MBS300723). Peroxidase-labelled secondary antibodies and 3, 3′-diaminobenzidine were applied to develop immunoreactivity, according to manufacturer’s protocol (EnVision; Dako, Glostrup, Denmark). The histopathological study by hematoxylin and eosin (H&E) staining was made independently by two pathologists (MJR and EDA). Ki67 labelling was quantified by ImageJ 1.45 s software.

### Statistical analysis

Mann–Whitney *U*-test for two groups, and one-way analysis of variance test for more than two groups followed by Tukey’s multiple comparisons post-test were used to evaluate differences between control and treatment conditions. The disease-free survival time was analyzed using the Kaplan–Meier estimator and the Wilcoxon test. For all analyses, *p*-values of ≤ 0.05 were considered statistically significant. Analyses were performed using the Prism 4.0 software (GraphPad). All experiments were carried out in triplicate.

## SUPPLEMENTARY MATERIALS FIGURES AND TABLES


