# Supplementary material for: The combination of epigenetic drugs SAHA and HCI-2509 synergistically inhibits EWS-FLI1 and tumor growth in Ewing sarcoma
Source: Oncotarget. 2018 Jul 31;9(59):31397–410. doi: 10.18632/oncotarget.25829 (PMC6101143; doi:10.18632/oncotarget.25829)
Supplement: Supplementary file 1 [file oncotarget-09-31397-s001.pdf]

# The combination of epigenetic drugs SAHA and HCI-2509 synergistically inhibits EWS-FLI1 and tumor growth in Ewing sarcoma

## SUPPLEMENTARY MATERIALS

**Supplementary Table 1: Taqman probes used for qRT-PCR**

| Genes | Brand | Reference | Amplicon length |
|---|---|---|---|
| CCND1 | Thermo Fisher | Hs00765553_m1 | 57 bp |
| DKK1 | Thermo Fisher | Hs00183740_m1 | 68 bp |
| EWSR1 | Thermo Fisher | Hs01580530_gH | 89 bp |
| EWS-FLI1 | Thermo Fisher | Hs03024497_ft | 80 bp |
| EZH2 | Thermo Fisher | Hs00544830_m1 | 86 bp |
| TGFβR2 | Thermo Fisher | Hs00234253_m1 | 70 bp |
| TPT1 | Thermo Fisher | Hs02621289_g1 | 131 bp |

**Supplementary Table 2: The clinical characteristics of the ES patients**

| Model code | Age at dx | Age at biopsy | Source of biopsy[1] | Primary tumor | Why biopsy | Fusion gene | STAG2 mutation | Patiet status[2] |
|---|---|---|---|---|---|---|---|---|
| HSJD-ES-001 | 17 y | 21.7 | Scapula | Scapula | Relapse | EWS-FLI1 type 2 | mutated | DOD |
| HSJD-ES-004 | 10 y | 18.0 | Mediastinum | Vertebral body T12 | Relapse | EWS-FLI1 type 1 | Wild type | NED |
| HSJD-ES-006 | 13 y | 13.7 | Lung nodule | Fibula | Relapse | EWS-FLI1 type 1 ex 10-ex 5 | Wild type | DOD |
| HSJD-ES-011 | 10 y | 13.9 | Pleura | n.a. | Relapse | EWS-FLI1 type 1 | Not done | AWD |

[1]Tumor tissues were collected with informed consent under an Institutional Review Board-approved protocol.
[2]DOD died of disease, NED no evidence of disease, AWD alive with disease.

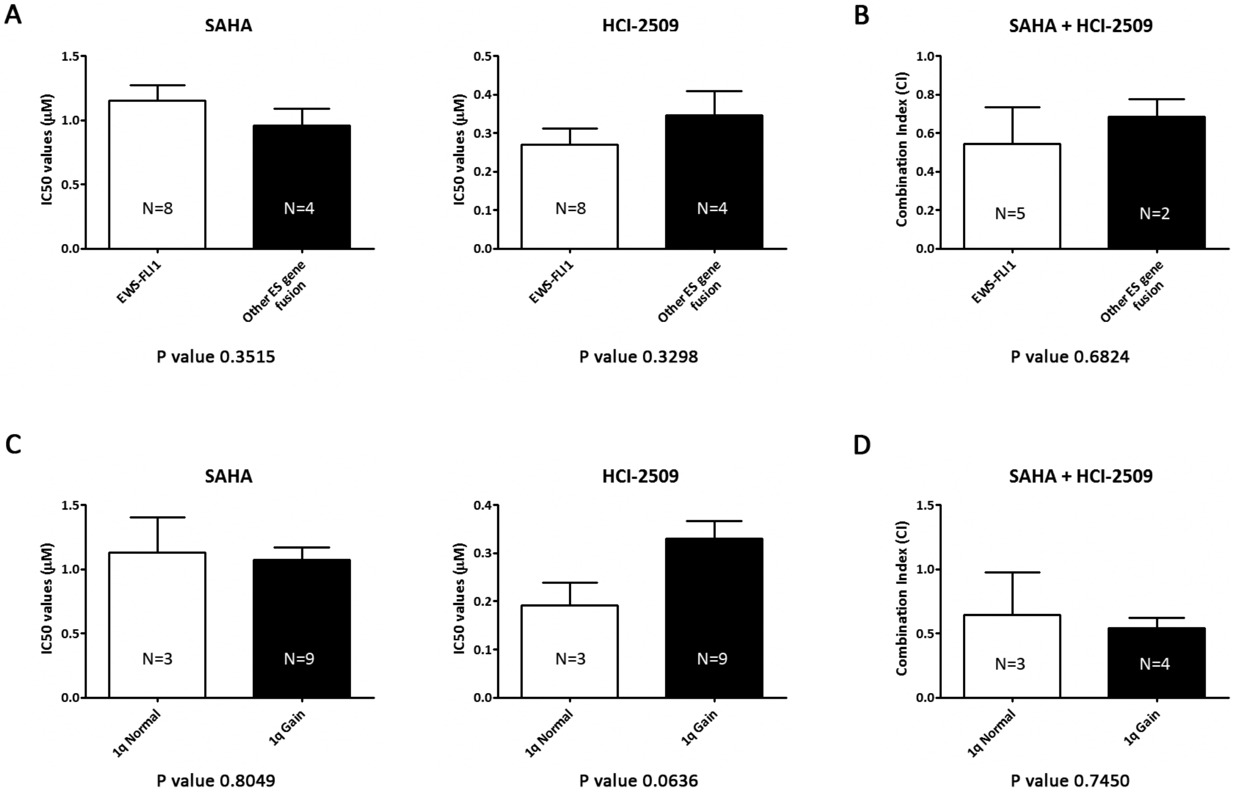


**E**

HCI-2509 and SAHA inhibitory concentrations

**HCI-2509 (iLSD1)**

| ***Cell Line*** | ***IC25 (µM)*** | *s.d.* | ***IC50 (µM)*** | *s.d.* | ***IC75 (µM)*** | *s.d.* | ***IC90 (µM)*** | *s.d.* |
|---|---|---|---|---|---|---|---|---|
| **A673** | 0.029 | 0.022 | 0.097 | 0.052 | 0.372 | 0.036 | 1.951 | 0.381 |
| **CADO-ES** | 0.166 | 0.027 | 0.282 | 0.039 | 0.494 | 0.042 | 0.877 | 0.111 |
| **TC32** | 0.117 | 0.032 | 0.187 | 0.019 | 0.479 | 0.323 | 0.780 | 0.193 |

**SAHA**

| ***Cell Line*** | ***IC25 (µM)*** | *s.d.* | ***IC50 (µM)*** | *s.d.* | ***IC75 (µM)*** | ***Cell Line*** | ***IC90 (µM)*** | *s.d.* |
|---|---|---|---|---|---|---|---|---|
| **A673** | 0.935 | 0.002 | 1.676 | 0.347 | 2.307 | 0.115 | 3.779 | 0.290 |
| **CADO-ES** | 0.604 | 0.118 | 1.089 | 0.168 | 1.634 | 0.286 | 2.755 | 0.568 |
| **TC32** | 0.902 | 0.123 | 1.553 | 0.160 | 2.495 | 0.253 | 4.432 | 0.505 |

Proliferation IC values of ES cell lines assayed for HCI-2509 or SAHA sensitivity and measured after 72h of drug exposition

**Supplementary Figure 1: Drug sensitivity is independent from ES fusion type or 1q status in ES cell lines.** (**A**) Mean of SAHA and HCI-2509 IC50 values between *EWS-FLI1* fusion type-ES cell lines group and others fusion types-ES cell line group (*EWS-ERG* and *EWS-FEV*) are shown. (**B**) SAHA+HCI-2509 combination index (CI) of *EWS-FLI1* fusion type-ES cell lines group and others fusion types-ES cell lines group is shown. (**C**) Drug sensitivity (IC50 values) between 1q normal ES cell lines group and 1q gain ES cell line group. (**D**) SAHA+HCI-2509 combination index (CI) of 1q normal ES cell lines group and 1q gain ES cell lines group. Values shown are mean ± s.d. of three independent replicates. (**E**) IC25, IC50, IC75 and IC90 values of ES cell lines assayed for HCI-2509 or SAHA after 72 h exposition to the drugs are shown.

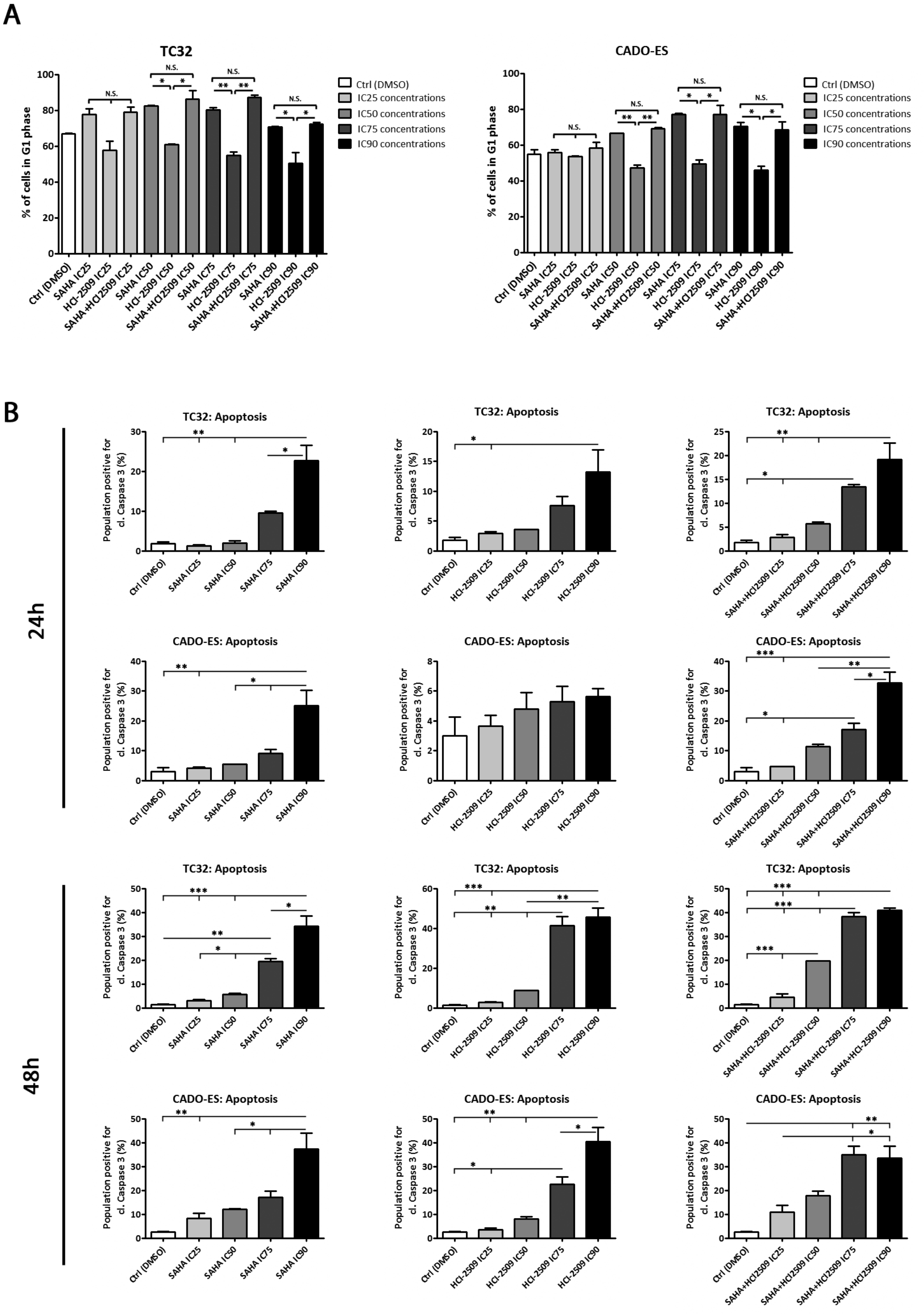


**Supplementary Figure 2: SAHA+HCI-2509 combination altered cell cycle progression and induced apoptosis.** (**A**) Quantification of cells in G1 phase of cell cycle after SAHA, HCI-2509 and combination treatment are shown in TC32 and CADO-ES cell lines. (**B**) Apoptosis induction detailed analysis of population positive for cleaved Caspase 3 in TC32 and CADO-ES cell lines after 24 h (upper panel) and 48 h (lower panel) of SAHA, HCI-2509, alone or in combination, at different concentrations. In all cases, mean ± s.d. of three biological independent replicates. Statistical tests: significant analysis of variance, Tukey post-hoc test <0.001 (***), 0.01 (**), and 0.05 (*).

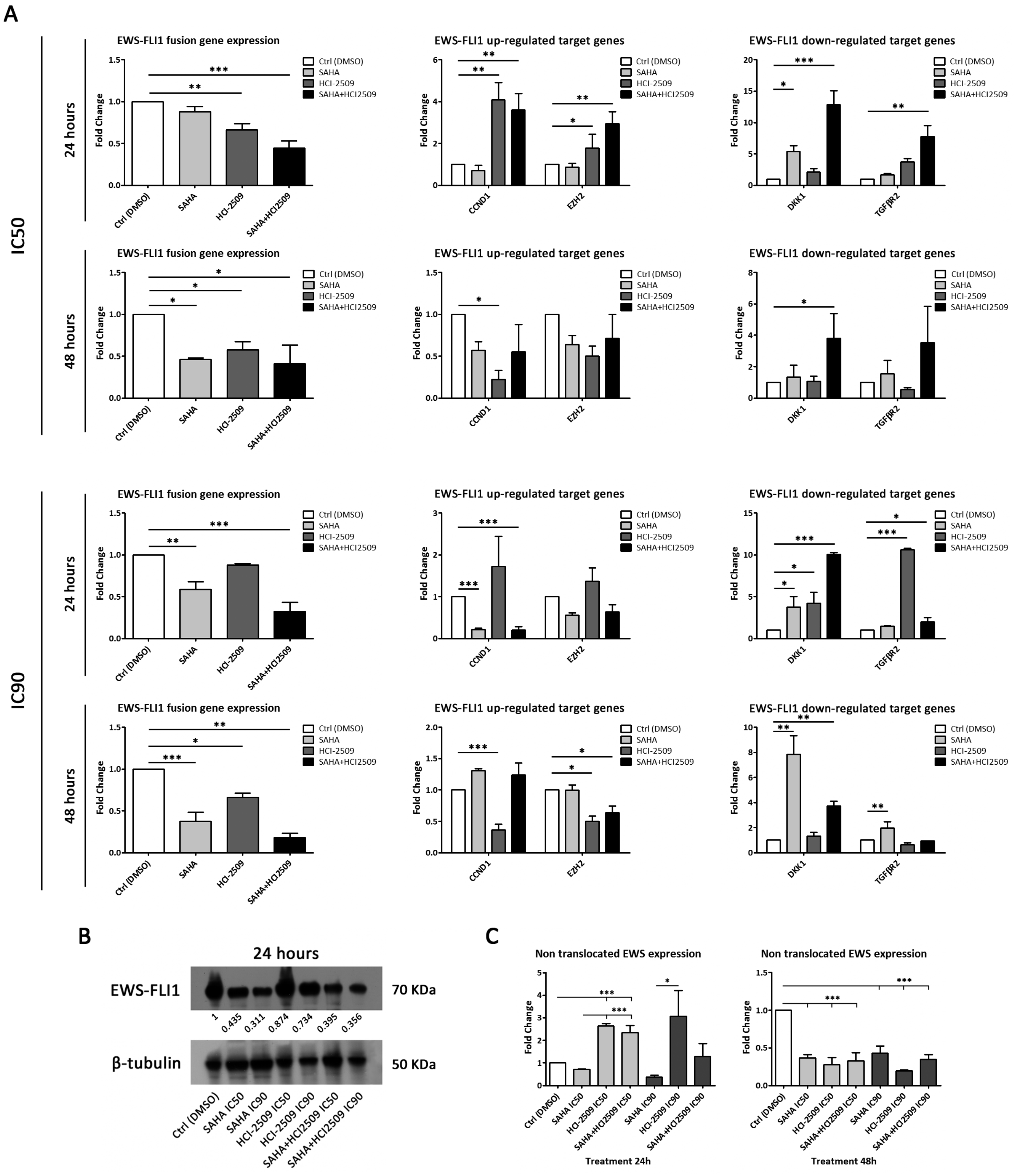


**Supplementary Figure 3: SAHA, HCI-2509, and SAHA+HCI-2509 treatment inhibited EWS-FLI1 expression in the A673 cell line.** (**A**) mRNA expression analysis by RT-qPCR of EWS-FLI1 (left column), and EWS-FLI1-induced/repressed target genes (middle/right column) after 24 h and 48 h of SAHA, HCI-2509 and combination treatment at IC50 (upper panel) and IC90 concentrations (lower panel) in A673 cell line. (**B**) Immunoblot of EWS-FLI1 protein expression after 24h of SAHA, HCI-2509 and combination treatment at IC50 and IC90 concentrations in A673 cell line is shown. Relative quantification is showed respect to the control (DMSO). (**C**) mRNA expression analysis by RT-qPCR of non-translocated EWSR1 after 24 h (left panel) and 48 h (right panel) of SAHA, HCI-2509 and combination treatment at IC50 and IC90 concentrations in TC32 cell line. Mean ± s.d. of three biological independent replicates is shown. Statistical tests: significant analysis of variance, Tukey post-hoc test <0.001 (***), 0.01 (**), and 0.05 (*).

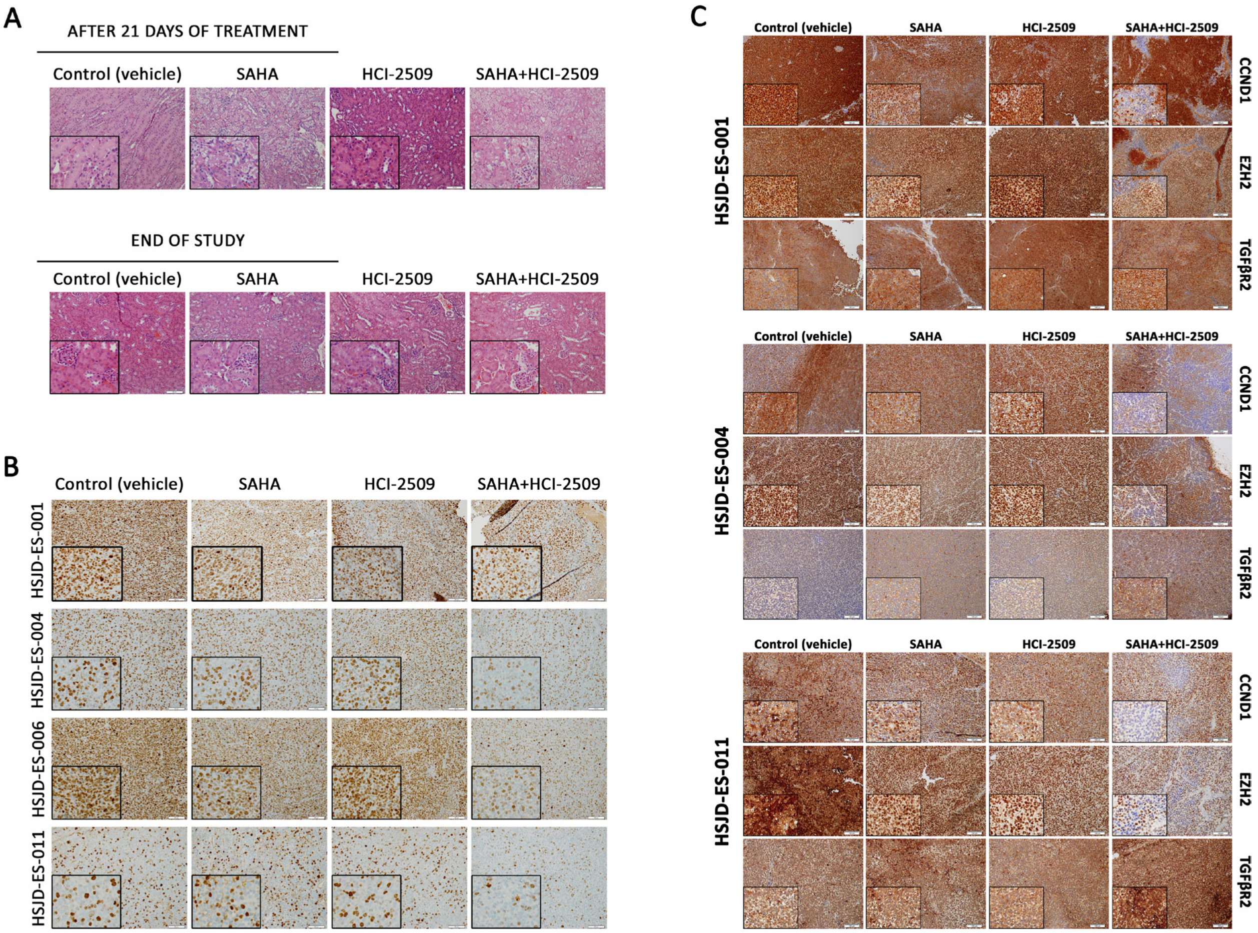


**Supplementary Figure 4: Histopathological study of PDX treated tumor samples.** (**A**) Morphological study of mice kidney tissue (hematoxylin and eosin staining) showed intracellular vacuolization induced by SAHA treatment in these samples. (**B**) Immunohistochemical staining of Ki67-positively labelled nuclei after 21 days of SAHA, HCI-2509, and combination treatment (20× and 40× magnifications). (**C**) Immunohistochemical staining of EWS-FLI1 targets CCND1, EZH2 and TGFβR2 in ES-PDX tumor samples treated in monotherapy or combination (20× and 40× magnifications).
